# The Effect of Environment and Husbandry Practices on Sheep Welfare

**DOI:** 10.3390/ani15223314

**Published:** 2025-11-17

**Authors:** Małgorzata Bąkowska, Renata Pilarczyk, Marta Juszczak-Czasnojć, Beata Seremak, Agnieszka Tomza-Marciniak, Ewa Kwita, Lidia Felska-Błaszczyk, Bogumiła Pilarczyk

**Affiliations:** 1 Department of Animal Reproduction Biotechnology and Environmental Hygiene, Faculty of Biotechnology and Animal Husbandry, West Pomeranian University of Technology in Szczecin, Klemensa Janickiego 29, 71-270 Szczecin, Poland; malgorzata.bakowska@zut.edu.pl (M.B.); beata.seremak@zut.edu.pl (B.S.); agnieszka.tomza-marciniak@zut.edu.pl (A.T.-M.); ewa.kwita@zut.edu.pl (E.K.); bogumila.pilarczyk@zut.edu.pl (B.P.); 2 Laboratory of Biostatistics, Bioinformatics and Animal Research, Faculty of Biotechnology and Animal Husbandry, West Pomeranian University of Technology in Szczecin, Klemensa Janickiego 29, 71-270 Szczecin, Poland; renata.pilarczyk@zut.edu.pl; 3 Department of Animal Anatomy and Zoology, West Pomeranian University of Technology in Szczecin, Klemensa Janickiego 33, 71-270 Szczecin, Poland; lidia.felska-blaszczyk@zut.edu.pl

**Keywords:** animal behaviour, disturbed behaviour in sheep, extensive husbandry, intensive husbandry, welfare, sheep–human interaction

## Abstract

The amount of attention paid to the welfare of animals and their maintenance conditions has grown considerably in recent years. This paper examines the effects of extensive and intensive housing systems on the environmental, behavioural, health, nutritional and social aspects of sheep welfare. It presents the current state of knowledge regarding this welfare, and its significant contributing factors, based on a search of PubMed, Web of Science, Google Scholar and Scopus using defined keywords. It compares the impact of the two systems on the physical, psychological and social comfort of the animals, and identifies husbandry practices conducive to improving welfare. In doing so, it pays particular attention to the role of social bonding and the importance of grooming interventions. The paper reviews the factors shaping welfare in different production systems, with a particular focus on extensive and intensive rearing. It demonstrates that while the extensive system is conducive to the realisation of natural behaviour and the formation of social bonds, it can also be associated with a higher risk of nutritional deficiencies and environmental stressors. In contrast, an intensive system allows better control of health and nutrition, but often limits the ability to meet behavioural needs. The study also discusses the importance of feeding strategies, herd structure and thermal comfort, as well as the impact of routines such as shearing and hoof correction. It demonstrates that high levels of welfare can be achieved in both extensive and intensive systems, provided that husbandry practices are appropriately adapted to the needs of sheep as a species.

## Simple Summary

Animal welfare has always played a key role in maintaining the physical and mental comfort of animals in their living environment; however, recent years have seen increasing attention paid to their welfare and living conditions. The article examines the effects of extensive and intensive rearing systems on the welfare of sheep, which is influenced by, inter alia, environmental conditions, diet, social interaction and human contact. The extensive system, based on grazing, favours the natural behaviour of the animals, but carries the risk of exposure to environmental factors and nutritional deficiencies. In contrast, the intensive system allows better control of health and production, but can have a negative impact on psychological welfare by limiting space and environmental stimuli. Nevertheless, measures can be implemented to mitigate these effects in both systems: welfare does not depend solely on the type of system, but also on the type and quality of husbandry practices, which should take into account the ethological and physiological needs of the animals.

## 1. Introduction

Animal welfare, defined as a state of complete physical, mental and behavioral well-being, is largely dependent on the ability of an organism to maintain homeostasis under changing environmental conditions [1]. An animal is best able to fully exploit its genetic and adaptive potential when its living conditions are suited to its biological needs; this forms the basis of the modern approach to keeping livestock. Under such conditions, the animals are less susceptible to diseases and injury, and those kept in environments that promote their natural behaviours demonstrate a higher standard of welfare [2,3,4,5].

Animal welfare is becoming an increasingly important consideration, especially in highly-developed countries with the knowledge, technology and financial resources to improve conditions for livestock. Welfare is also an important element of European Union policy, and is recognised as a cornerstone of sustainable development in the European Green Deal. The increasing importance attributed to animal welfare in farming practice is due not only to ethical considerations, but also to the growing awareness that the comfort of animals directly correlates with their health, reproduction and the quality of the products obtained.

Sheep often demonstrate ambiguous behavioural responses and are hence considered ‘behaviourally enigmatic’; as a result, it can sometimes be difficult to assess their welfare. Even so, many sheep are kept in an extensive system with access to pasture, which allows them to move freely, forage and engage in natural social interaction. Such animals are at a lower risk of manifesting stereotypic behaviour, which is often a symptom of chronic stress and ill-being.

While extensive systems are considered to be among the more animal-friendly housing systems, they can still present serious risks to animal health and welfare. Under conditions of drought or poor pasture quality, sheep can be exposed to starvation, nutritional deficiencies and stress from extreme weather conditions such as heavy rain, wind or extreme temperatures. This risk is exacerbated by a lack of adequate shelter. In addition, the presence of herding dogs can lead to increased stress, especially if their interactions are more intense or aggressive [6].

Nevertheless, Nedeva [7] proposes that, despite these greater environmental risks, extensive systems respond better to the biological needs of the sheep. In contrast, the intensive system allows precise control of health and production, but limits the freedom and natural behaviour of the animals. The extensive system favours the fulfilment of natural behavioural and social needs, which can translate into higher welfare. At the same time, it is associated with greater exposure to environmental factors and difficulties in ensuring stable nutritional and health conditions. In contrast, an intensive system, despite better control over microclimate, nutrition and health, limits space, stimuli and spontaneous social interactions, which can lead to frustration, stress and increased risk of behavioural disorders [6,7,8,9].

While sheep welfare has been the subject of numerous studies to date, there remains a lack of integrated analyses comparing the impact of extensive and intensive farming systems on the major dimensions of their welfare, these being their physiological, behavioural, environmental and social aspects. Sheep welfare is known to be subject to a range of influences, including nutritional conditions, thermal comfort, hygiene, access to water and shelter, health, quality of care, human–animal relationships and herd social structure. It is essential to understand the interactions between these factors and the husbandry system to ensure a high standard of welfare.

Animal welfare in general can be comprehensively assessed using a range of protocols and indicators. Some are based on observations of animal behaviour and physiological condition (i.e., animal-based indicators), while others examine environmental conditions and resources. Each one, such as AWIN, Napolitano, Stubsjøen and Munoz, uses a different set of indicators, thus allowing welfare to be assessed in both intensive and extensive systems and the identification of potential problems.

The aim of this study is to provide an up-to-date review of the literature on sheep welfare, with a particular emphasis on the factors affecting welfare and assessment indicators and protocols used in different husbandry systems. It aims to organise and compare existing methodologies and provide integrated knowledge on the subject. The guiding hypothesis is that different sheep husbandry systems affect various aspects of welfare to different degrees, and these differences can be best determined by combining animal-based indicators and environmental parameters. The results of the review may serve as a starting point for the development of consistent welfare assessment standards and potential breeding recommendations. As such, its findings are intended to add new value to existing literature on the subject.

## 2. Materials and Methods

The work presents a comprehensive analysis of the factors influencing sheep welfare in different housing systems, with a focus on the environmental, behavioural, health, nutritional and social aspects. It compares the impact of extensive and intensive housing systems on the physical, psychological and social well-being of sheep, and identifies husbandry practices that can enhance their welfare it also discusses the importance of social bonding, handling and human–animal interactions, and the impact of husbandry practices, and welfare associated with transport, slaughter or humane killing, on overall welfare.

Selected scientific databases, i.e., PubMed, Web of Science, Google Scholar and Scopus, were searched for information on the current state of knowledge regarding sheep (*Ovis aries*) welfare. The searches were based on the following keywords: “sheep welfare”, “Ovis aries behavior”, “sheep health indicators”, “environmental enrichment for sheep”, “sheep–human interaction”, “sheep social behavior”, “stress indicators in sheep”, “aberrant behavior in sheep”, “thermoregulation in sheep”, “behavioral indicators of welfare in sheep”, “animal-based welfare indicators”, “ethological needs of sheep”, “body condition score (BCS)”, “lameness in sheep”, “fleece condition”, “tail length and dag score”, “welfare assessment protocols”, “emotional well-being of sheep”, “maternal and lamb welfare”, “handling and human–animal interactions”, “transport welfare”, “slaughter welfare/humane killing”. These phrases were linked with the logical operators “AND” and “OR” in various configurations, such as “sheep welfare” OR “Ovis aries behavior”. In order to limit the results to species-specific studies, the “AND” operator was sometimes added together with the word sheep. The syntax of each query was adapted to fit the specifics of each database and its advanced search tools.

**Figure 1:**
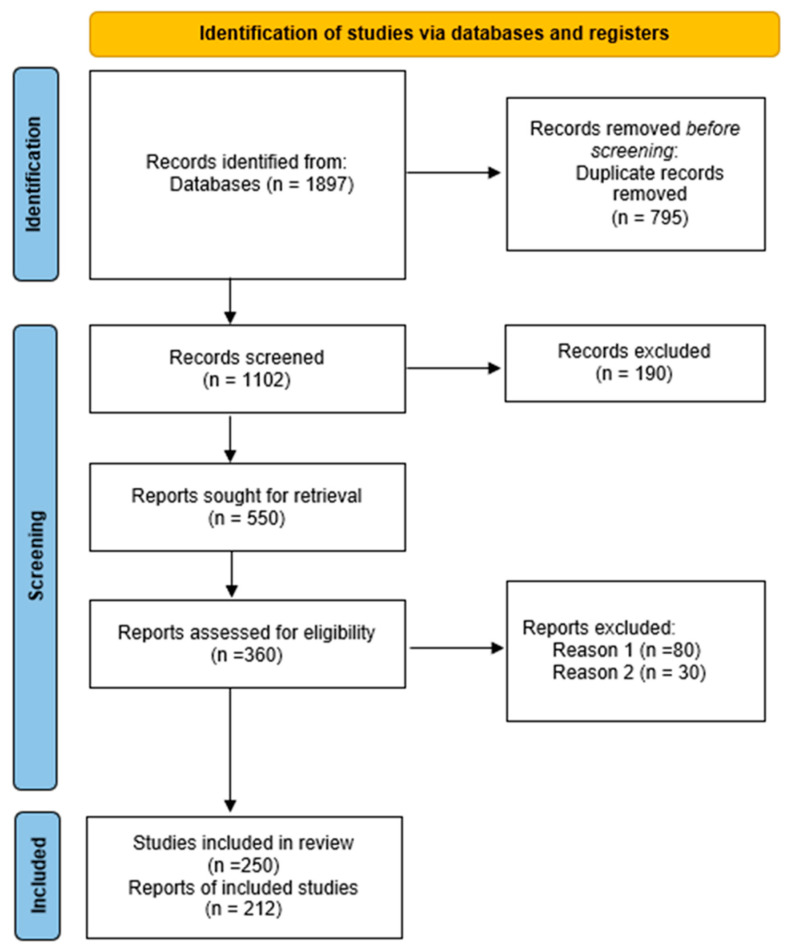
Flowchart of the process of identifying and selecting publications for the literature review based on Page et al. [10].

The collected material included original articles, literature reviews, books and industry reports. Most of the material was identified in Google Scholar; however, a significant proportion of publications were excluded due to them being of limited scientific value, such as preprints and theses. In total, the search returned 1897 original results. After removing duplicates, 1102 unique publications remained. Of these, 550 papers were selected based on a review of the titles and abstracts. Of these, 190 were rejected as being unrelated to the topic. After these were eliminated, 360 publications remained; 80 of these were not available in full text and were rejected, and another 30 were rejected for methodological reasons. Ultimately, 250 papers were selected for further analysis, of which 212 key items were selected for detailed review (Figure 1).

The included publications concerned sheep welfare or related aspects of breeding, behaviour, feeding, social relations, human contact, grooming, training, welfare of mothers and lambs, handling and human–animal interactions, transport welfare, slaughter welfare/humane killing, or environmental enrichment. These included original research, literature reviews and chapters providing data. Papers lacking access to the full text were excluded, as no thorough assessment of the methodology and results was possible.

The selected articles are divided thematically into the following areas: welfare of sheep under breeding conditions, effects of nutrition on welfare, human–sheep relations, social and reproductive behaviour, the mother–offspring bond, welfare during grooming, effects of environmental enrichment on sheep behaviour, handling and human–animal interactions, transport welfare, slaughter welfare/humane killing, and indicators of stress and welfare. The collected data was then systematised and presented in the following sections of the article, allowing for a comprehensive discussion of the issue of sheep welfare.

## 3. Relevant Sections

### 3.1. Habitat

In an extensive system, sheep are kept in open, natural pastures; this provides the animals with freedom of movement, access to a diverse environment and the opportunity to perform natural behaviours such as exploring, foraging and forming social bonds. Such conditions promote higher behavioural well-being and a more stable social hierarchy [6,7]. The intensive system, on the other hand, is characterised by limited space (often 1–2 m^2^ per sheep). The animals are kept in enclosed or semi-enclosed buildings with a controlled microclimate, which increases production efficiency but significantly reduces natural stimuli and freedom of movement [8,9,11] (Table 1).

For nutrition, the extensive system mainly uses roughage (grasses, hay), whose quality and availability depend on the local climate. For example, the nutritional value of the pasture sward can decline during the summer season, especially during periods of drought, increasing the risk of nutritional deficiencies and stress [7]. In addition, the sheep are at a higher risk of infestation by internal parasites, such as gastrointestinal nematodes. In contrast, in the intensive system, feeding is carefully controlled, relying on concentrate mixtures and silage; while this allows a better balance of rations, it can promote metabolic diseases if not properly managed [7,11] (Table 1).

The two systems also have different effects on social relationships. In extensive rearing, sheep form more natural social structures, favouring stable hierarchies and lower levels of aggression. It is also possible to form strong mother–offspring bonds and spontaneously create social subgroups. The greater space afforded each animal allows individual distances to be maintained and conflicts to be avoided. In the intensive system, higher animal densities and frequent mixing of groups are observed, which disrupts social relationships, increases agonistic interactions and can lead to behavioural frustration [6,7,9,11] (Table 1).

In the intensive system, the sheep are in daily contact with humans as part of a range of activities, such as feeding, milking or zootechnical and veterinary procedures. This can lead to stress, especially if the interactions are inappropriate. In an extensive system, on the other hand, the contact is limited to periodic treatments (e.g., shearing, treatment). While this may reduce the stress associated with the presence of humans, it can also mean that sudden intervention may be perceived by the animal as a threat [7] (Table 1).

The systems also differ in terms of health aspects. In the intensive system, despite better control of feeding and prophylaxis, high animal density increases the risk of rapid spread of infectious diseases such as mastitis and lameness. While this spread of infection is more limited in the extensive system due to the lower stocking density, the sheep are at a greater risk of parasitic infections and are more exposed to environmental factors such as weather conditions [6,7] (Table 1).

The two systems also influence reproductive behaviour. While the extensive system favours natural mate choice and spontaneous sexual behaviour, the intensive system usually controls reproduction (e.g., by synchronised oestrus and artificial insemination), which can interfere with the natural course of the sexual cycle and cause frustration [7,9]. As a result, extensive systems tend to have greater social welfare because the sheep are better able to take part in their natural interactions and behaviours. Although extensive systems favour natural mate selection and spontaneous reproductive behaviour, they employ various methods of controlling the reproductive cycle, such as teaser rams or melatonin implants, with the aim of synchronising oestrus for breeding. However, exposing ewes to male sexual stimuli without the possibility of copulation increases their sexual motivation; this can increase frustration and stress, manifested by, inter alia, elevated LH, testosterone and cortisol levels, as well as homeostasis and reproductive cycle disruption [12]. Alternatively, hormone-induced oestrus synchronisation can dramatically increase sexual activity [13], resulting in more intense competition between males and greater stress in females. In addition, artificial insemination limits the possibility of natural reproductive behaviour, which may also result in signs of behavioural frustration, such as increased vocalisation and agitation [14]. Such disruptions of the natural behaviour and hormonal rhythms of animals have been identified in FAWC reports as potential risk factors for their welfare [15]. Hence, the true level of welfare on farms is determined by the type and scale of the employed reproductive intervention, and the manner of their implementation (Table 1).

The animals also demonstrate different responses to environmental changes. In the extensive system, the animals have to respond to changing conditions with more adaptive flexibility; while this may develop their adaptability, it can also be associated with stress, such as when being chased. The intensive system is characterised by more stable and predictable environmental conditions, but prolonged exposure to monotony can also limit adaptability [8] (Table 1).

Herd movement can be coordinated in different ways depending on the type of system. In extensive systems, herd movement is largely spontaneous and often initiated by the so-called leader, i.e., the animal that sets off first and, in a sense, leads the rest of the group. Interestingly, the leader does not have to be a dominant individual: in some herds, younger animals and even lambs have been found to take the initiative when moving. In contrast, in intensive systems, animal movement is usually planned and supervised by humans and conditioned by farm infrastructure (e.g., corridors, gates), which limits the possibility of natural behaviour and can lead to tension within the herd. While movement is influenced by humans in both systems, the degree and manner of this influence differ between the two [16,17].

Unfavourable housing conditions adversely affect sheep behaviour, which can represent a clear sign of deterioration in welfare [18,19]. Lambs housed in environments lacking adequate stimuli can demonstrate elevated cortisol levels and a higher risk of stereotypic behaviours such as bar biting, which has been attributed to chronic stress [20]. Failure to fulfil natural behavioural needs such as exploration or chewing also leads to behavioural disorders and is a sign of reduced well-being.

The environment can be effectively enriched by the introduction of bedding, especially in the form of straw, and this can significantly promote the welfare of lambs. The presence of bedding promotes exploratory behaviour, reduces hypersensitivity to new stimuli and reduces stereotypic behaviour [21]. In addition, it improves thermal comfort and allows the sheep to chew, which can ease reactivity to environmental stressors such as transport or group change. Teixeira et al. [20] confirm that lambs kept on litter show lower levels of aggression and more frequent positive social interactions.

While the extensive system encourages natural behaviour, the intensive system allows better control of nutrition and health. Nevertheless, in both cases, conscious management and appropriate practices are needed to ensure a high level of welfare among the sheep (Table 2).

#### 3.1.1. Air Temperature and Humidity

Being ruminants, sheep show relatively high resistance to extreme environmental conditions, and can survive for longer periods without access to food and water than other livestock species [31]. Even so, despite this adaptive resilience, sheep are still at risk of significant health impairments, reduced production efficiency and poorer overall welfare following prolonged exposure to excessive heat. Heat stress, beyond the limits of heat tolerance, leads to reduced feed intake, reduced milk yield, dehydration and increased risk of disease [32].

When encountering heat stress, sheep can engage in a number of behavioural and physiological–biochemical adaptive mechanisms. They may seek shaded areas or increase their water intake, and their organisms activate responses that enable efficient thermoregulation. These strategies, although necessary for survival, often come at the expense of other behavioural needs, such as exploration, foraging or social interaction, which can reduce animal welfare in the long term. It is also important that they are provided with appropriate environmental conditions, such as good ventilation, access to shade or the presence of cool resting areas, which support natural adaptation mechanisms and contribute to their welfare [32].

When the thermal tolerance threshold is exceeded, sheep can experience serious health problems, such as dehydration, digestive disorders or loss of appetite, which directly translates into a decrease in milk production. Persistent thermal discomfort and physiological disturbances further reduce animal welfare by inducing chronic stress, which impairs immune system function, disrupts metabolic balance and worsens overall body condition [32].

In some environments, sheep kept in closed systems may lack access to air conditioning or an adequate cooling system. In such cases high temperatures cause marked changes in both behaviour and body function. The animals typically demonstrate reduced locomotor activity, spending more time lying down and less time foraging and moving around. These activities are associated with increased respiratory and heart rates, which are intended to regulate body temperature. The animals also exhibit elevated internal temperature, indicating increased heat stress. Heat stress is also associated with elevated cortisol levels and disturbances in the electrolyte and metabolic balance. However, the response to heat is influenced by both individual and sex factors: females (ewes) tend to show greater resistance to high temperatures than males (rams) [33].

Prolonged exposure to high temperatures increases the risk of disturbances in homeostasis, changes in behaviour, reduced feed intake, and deterioration of physiological and metabolic parameters. These in turn can lower production efficiency, reduce the quality of animal products and impair reproductive performance. Importantly, the negative effects of heat stress are exacerbated by high relative humidity; by reducing the efficiency of evaporative thermoregulation, the sheep are unable to cool down [34,35].

During harsh weather conditions, including high temperatures, it is extremely important to create a suitable housing environment for sheep which should be designed to limit the impact of heat stress. The effectiveness of the environment is influenced, inter alia, by the construction materials, the provision of shaded areas, the efficiency of air circulation and access to daylight. In the summer months in particular, it is important to ensure that the animals have easy access to fresh water and areas sheltered from the sun [36].

#### 3.1.2. Nutrition

To address their nutritional needs, a sheep housing system should offer access to farm-grown fodder, particularly the use of pasture, for as long as possible; pasture grass is the most natural feed for sheep and its constant availability promotes animal welfare. In extensive systems, the sheep are able to select plants themselves, guided by their senses [37], and to explore their environment freely. They are also able to associate the sensory characteristics of food, such as taste or smell, with its effects after consumption and adjust their choices accordingly [38]. It is recommended that roughage makes up at least 60% of the dry matter of the daily ration. Uninterrupted access to water is also very important.

In Poland, in response to economic pressures and the animal welfare legal environment, the two most commonly used housing systems in sheep farming are extensive and semi-intensive. The extensive system is based mainly on pasture grazing, which significantly reduces feeding costs while allowing the animals freedom of movement and access to fresh feed. During the winter and transitional periods, the sheep are housed in simple, functional enclosures adapted to their needs [39].

Semi-intensive rearing, on the other hand, combines grazing with additional concentrated feeds, allowing better production results while maintaining near-natural conditions. Both systems respond well to the Polish climate and economic conditions, allowing a compromise between profitability and animal care [39].

Sheep form hierarchical groups in which lower-ranking individuals may be pushed away from feeders and drinkers. As a result, animals of lower social status have to spend more time and energy to gain a place at the feedlot or drinker. Therefore, if access to food and water is not available simultaneously for the whole flock, some sheep may not consume adequate feed, reducing their growth and welfare [37].

Correct feed regimens should not only fully cover the nutritional requirements of the animals, but the ration or complete mix should be in a suitable form. When feeding sheep, it is crucial to maintain a correct ratio between finely-ground and that natural feed. Excessive fineness can disrupt the function of the foregut, leading to digestive problems and reduced animal performance. As previously recommended, in adult sheep, 60% of the daily ration should be roughage. Such balanced feeding promotes proper fermentation in the rumen, ensures adequate filling of the digestive tract and promotes good condition and high productivity of the flock. An excess of finely-ground ingredients hampers digestion, reduces saliva production and can lead to stomach problems such as bloat [37,39,40,41].

When fattening lambs, if grain mixtures are used, at least half of the grain should be fed as whole or coarsely crushed grains, rather than finely ground. For complete feeding mixtures, it is helpful to add a small amount of hay (70–100 g per kilogram of feed): this provides an appropriate feed structure, promotes digestion and foregut development, and improves the well-being of the animals by increasing satiety [39,42].

Under the semi-intensive system, lambs are kept with their mothers throughout the rearing and fattening period. The mothers are housed under maximally extensive conditions in winter, in buildings with access to circular areas during transitional periods, and mainly on pasture in summer. The lambs receive more intensive feeding, allowing for more efficient weight gain. This approach reduces the stress of separating the offspring from their mothers, which is particularly important during the strong bonding period. The presence of the mother also allows her milk to be fully utilised as a natural food. This combination of shared rearing and providing access to pasture has been found to improve both animal welfare and lamb meat quality [39].

Intensively fattening lambs after separation from their mothers is less beneficial to their welfare than fattening them with their mothers; however, a properly managed weaning process can significantly reduce the stress involved [39].

#### 3.1.3. Weaning Lambs

One of the most stressful procedures in sheep farming is weaning the lambs from their mothers, a procedure that can be both psychologically and physiologically stressful; stress in animals induces specific behavioural, physiological and hormonal responses that can significantly affect their welfare [43]. In the case of lambs, weaning not only disrupts the strong maternal bond [44], but also requires sudden adaptation to a new diet, placing a double burden on the young organisms.

The response of lambs to weaning appears to be influenced by several factors [44]. Firstly, a significant role is played by weaning age: younger lambs, which share a stronger bond with the mother, show more intense stress reactions than older individuals [44]. Stress is also influenced by weaning method: Orgeur et al. [45] note that lambs subjected to complete separation from their mothers showed higher cortisol levels than those that had retained visual and auditory contact.

Behavioural responses to weaning are particularly pronounced. Many young animals demonstrate increased vocalisation following separation from their mothers [46]. Mears and Brown [47] add that lambs separated from their mothers express their stress through intense burping. Importantly, a study by Schichowski et al. [44] found that lambs weaned gradually, i.e., with a one-week period of restricted access to milk before full separation, exhibited less stress behaviour than those experiencing traditional, abrupt weaning.

At the physiological level, weaning induces a number of changes. Napolitano et al. [48] found that mother-weaned lambs demonstrated a lower cortisol response compared to those which were artificially fed. In contrast, Ekiz et al. [49] report increased plasma cortisol concentrations in lambs weaned at both 45 and 75 days of age.

### 3.2. Care Treatments

The simplest method of determining the welfare of animals during grooming is to measure the level of stress they experience. By analysing these stress responses, it is possible to understand how animals experience the treatment [50].

#### 3.2.1. Shearing

One basic procedure performed as part of sheep husbandry is shearing. Its main purpose is to obtain wool, but it also improves resistance to high summer temperatures and reduces hygiene problems [51]. Studies on Akkaraman sheep suggest that they cope better with high temperatures after shearing, and that the procedure affects various physiological and hormonal parameters, such as body weight, rectal temperature, heart rate, cortisol levels, β-endorphins, growth hormone and thyroid hormone levels [52].The date of shearing is based on both the physiological state of the animals and the wool quality requirements. In the case of rams, this treatment is usually carried out a few weeks before the breeding season: the loss of the fleece promotes an increase in sexual activity and improves the appearance of the wool, which grows at this time, thus improving its commercial value [53]. Shearing should also be carried out before stressful events, i.e., before the final stages of gestation, lambing and lactation: these stages are typically associated with fibre constriction, i.e., a reduction in fibre diameter (so-called wool break). This significantly compromises the quality of the fleece, especially when the weakened area is in the middle part of the hair, preventing its use in textiles.

The timing of shearing has also been found to significantly affect thermal comfort and the quality and quantity of milk produced [53]. When shearing was carried out in late gestation, i.e., around day 100, the animals tolerated high temperatures better and their physiological responses were noticeably milder than ewes sheared earlier or remaining in fleece. Importantly, late shearing did not adversely affect the reproductive or rearing ability, and no differences were observed in lamb birth weight, growth in the first weeks of life, colostrum quality or overall milk yield. In addition, the sheep were found to be more productive with improved milk composition, i.e., increased protein, fat and casein content. In the Lacaune breed, total milk production in the subsequent lactation phases was found to increase by up to 28% [53]. Shearing is also easier before parturition, when the skin is taut and the procedure easier to perform: after weaning, the procedure becomes more difficult, more time-consuming and results in greater wool loss.

In the UK, industry guidelines indicate that shearing should be performed on dry ewes because they are calmer, easier to handle and less prone to injury: lactation causes the milk veins to enlarge, which increases the risk of cuts. Furthermore, in pregnant ewes, the heavy belly complicates shearing, which can cause stress and threaten animal welfare. Therefore, it is recommended that ewes should not be sheared sooner than eight to twelve weeks after lambing [54].

Although the shearing procedure has a number of benefits, it can nevertheless cause severe short-term stress [50,55]. The sheep demonstrate behavioural changes, and alterations in autonomic nervous, endocrine and immune system functioning [50,55,56,57]. The intensity of the response depends on both the type of stimulus, and on personal characteristics, such as temperament or stress sensitivity [58,59].

The effect of shearing on thermoregulation depends on breed and climate. For example, it has been found to improve temperature regulation in Akkaraman sheep [52], but not to demonstrate any significant improvement in crossbreeds adapted to warm climates, suggesting that their natural thermoregulatory mechanisms are sufficient [60].

While the shearing process has been found to cause stress, no significant differences in stress indicators, i.e., MDA, GSH, cortisol, T3 or T4 level, were noted between the groups sheared with a machine shearing or with hand shears. Both groups demonstrated clear changes in all measured stress parameters during the procedure, indicating that shearing had a significant effect on the animals. Shearing is considered to be one of the most stressful routine procedures in sheep management, with level of stress experienced being dependent on previous experience with a particular shearing method [50].

#### 3.2.2. Hoof Correction

Hoof correction is mainly performed in areas where the animals are kept on soft ground, with the frequency of correction depending on the rate at which the hooves wear down. In many countries, including Poland, this procedure is usually performed twice a year, i.e., in spring and summer. In places where hooves wear down naturally, or where rot occurs, this procedure is performed when needed; however, performing routine hoof correction more than once a year has been found to increase the risk of footrot [61]. Generally speaking, in some countries, there is a need to review the approach to preventing and treating foot disease in sheep.

Hoof correction can induce a significant stress response, whose nature and severity are closely dependent on the handling method. Yardimci et al. [50] report clear differences in the stress response of sheep depending on the method of hoof correction. Sheep maintained in a standing position demonstrated a mean cortisol level of 17.39 ng/mL, with this value being twice as low as those treated using the traditional method, i.e., lying down (34.35 ng/mL). Lying down also appears to place a greater burden on the body, with higher MDA levels being observed (5.38 nmol/mL) compared to standing (4.14 nmol/mL), suggesting a stronger oxidative response, and slightly elevated GSH levels (11.65 vs. 11.00 mg/dL). This may reflect enhanced mobilisation of defence mechanisms. Acute, short-term stress is associated with an increase in chemotaxis and particle adhesion, which stimulates the migration of immune cells to sites of infection or inflammation. This can be positive, as it results in greater resistance to infections, such as leg disease in sheep [62].

### 3.3. Social Bonds

In addition to their physical well-being, the living environment also plays a key role in shaping sheep social behaviour and flock structure. Sheep are herd animals, and as such have a strong need to be in a group, pay constant vigilance to their surroundings and interact freely with other flock members [63,64]. Females form lasting social relationships, as evidenced by Dumont and Boissy [65] and Fisher and Matthews [66].

Sheep show great flexibility in their habitat choice and adapt their spatial behaviour to changing weather conditions and seasons. Their environmental preferences are strongly influenced by the availability of food, and the need to limit energy loss to maintain a suitable body temperature [64].

#### 3.3.1. The Effect of Climate Conditions on Social Behaviour

Doyle et al. [64] report that, in addition to age, social behaviour in the flock is also influenced by air temperature and the amount of precipitation.

Higher air temperatures have been found to favour longer contact times between sheep. In one study, sheep spent an average of 34 min 40 s with each other on hot days, compared to only 18 min 17 s on the coolest days. These differences can be accounted for by the tendency to congregate in the shade, where it is easier to maintain proximity. Similarly, rainfall caused the sheep to stay closer together: during the wettest days, contact lasted more than half an hour, shortening to around 10 min 32 s in the absence of rainfall. In heavy and prolonged precipitation, sheep tend to stop grazing, reduce locomotor activity and seek shelter [67].

#### 3.3.2. The Influence of Food Availability and Group Size on Social Behaviour

Hierarchy also influences feeding behaviour. During alcove feeding, dominant individuals have priority at the feeders. When space is scarce, competition can be reduced by using a baffle feeding system to separate groups and reduce pressure [68].

Dumont and Boissy [65] report that sheep prefer to be with others, even if this meant foraging on less attractive grass. When quality food was close to the flock (e.g., 15 m away), the sheep used it regardless of group size. However, when good food was further away (e.g., 50 m from the flock), sheep tended to stay close to the group; an exception was noted for larger groups of about seven individuals, who felt safe enough to move away from the flock.

Group size had a significant effect on behaviour: small groups, i.e., one to three sheep, were more cautious, spent more time on alertness (raising their heads, listening) and stayed close to the flock, even at the expense of inferior food. Larger groups comprising four to seven sheep explored the area more boldly, moved away more often to reach better grass, feeling safer and showing fewer signs of vigilance [65].

#### 3.3.3. Social Hierarchy and Flock Behaviour

Social hierarchy plays a very important role in maintaining order and reducing conflicts between individuals. Each sheep recognises its own position in relation to other group members; this makes it possible to avoid confrontation and facilitates coexistence within the flock [69]. Most rivalry occurs between individuals of similar social status.

Kiełtyka-Kurc and Górecki [70] note that various factors, such as sex and degree of relatedness, can shape social relationships between lambs. Hierarchy rules also apply during resting. Younger lambs, being the lowest in the social structure, tend to occupy the least comfortable places. The introduction of a new individual into a stabilised group involves a temporary disruption of the hierarchy. The integration process can take from a few weeks to even a few months; after this, a new set of relationships is established, reducing the frequency of conflicts [69]. Violating the individual space of another sheep results only in a warning signal, i.e., a threat, resulting in the lower status individual giving way [71].

The hierarchical system is formed by direct interactions between individuals, including agonistic behaviour such as threatening or hitting. Age is important in the social structure, usually with the oldest female acting as a leader and younger individuals learning appropriate behaviour from her. In practice, this means that young ewes are more likely to follow experienced mothers [69].

Sheep of similar age spend more time together (approximately 20 min 43 s per day) than individuals with a large age difference (16 min 33 s). This preference may be due to hierarchy in the flock or similar behavioural patterns [64].

Social bonds in sheep are very strong and play a very important role in their flocking behaviour. Indeed, sheep prefer to remain close to their companions, even in situations where moving away from the group could provide an advantage, such as access to better food [65]. This effect is even stronger when individuals have known each other since they were young, indicating that familiarity and attachment reinforce their tendency to stay together [16].

Furthermore, social bonds and individual independence appear to influence spontaneous movements of groups of sheep to a greater degree than dominance or temperament. Individuals that are strongly bonded to each other are more likely to move together [16].

Interestingly, sheep with similar vocalisation frequencies have been found to spend more time with each other (27 min 16 s) than those with different levels of vocalisation (19 min 36 s; [64]). This fact suggests that similarities in communication promote the building and maintenance of social bonds in the flock.

#### 3.3.4. Formation of Mother–Offspring Bonds in Sheep

The formation of the bond between ewe and lamb is a complex multi-sensory process that is plays an important part in the survival of the offspring. Of particular importance is the period of receptivity, which occurs immediately after birth. During this time, the mother learns to recognise her young primarily by smell, this being the dominant sense responsible for selective acceptance at udder; this cue enables the lamb to be recognised as early as approximately four hours after birth [72]. After birth, the lamb needs to get up quickly. i.e., within 30 min, find the udder and start suckling, which usually takes place after one to two hours. Lambs explore the area around the mother’s underbelly, guided by warmth, touch and smell. Sheep often facilitate their access to milk by changing their body position. Through close interaction and suckling, lambs quickly learn the location of the teats. They prefer warm, smooth surfaces with moderate softness [73,74]. Suckling helps lambs calm down and helps them absorb new information [74]. Over the following hours, the scent is supplemented by other visual and auditory stimuli; these allow the mother to identify the lamb from a distance, which is observed around 12 h after birth [72].

A key role in the sustained recognition of offspring is played by imprinting; this is made possible by intensive sensory stimulation and neurohormonal activation of the brain structures responsible for maternal behaviour, such as the hypothalamus and amygdala [74]. Social bonding is also facilitated by oxytocin: a hormone that stimulates mothers to care for their offspring by promoting tactile contact such as licking and feeding, which supports the development of the lamb [75]. Oxytocin has also been found to affect memory capacity and brain plasticity in female sheep during gestation and lactation, which promotes the maintenance of caring behaviour [76].

During the first weeks of life, the mother significantly increases the chances of survival of the offspring by providing food, warmth and protection [77]. Over time, as the lambs mature, feeding becomes less frequent [78] and the young begin to explore their environment independently and develop social relationships [79].

The quality of the mother-lamb bond is also influenced by, inter alia, breed, number of previous births and maternal nutritional status [80]. Malnourishment, particularly when the sheep is kept on pasture during drought conditions, can result in abnormal gestation, impairing both foetal development and maternal behaviour [81,82]. These effects are more pronounced in bighorn sheep, which require more energy giving birth to larger lambs [83].

Maternal behaviour also varies according to previous experience of raising young. Primiparous ewes often show weaker maternal instincts: their lambs tend to be lighter, stand up later and start suckling, and the mothers themselves show less behavioural stability when interacting with their young [84,85]. Furthermore, even 24 h after birth, these lambs may have difficulty recognising the mother, significantly increasing the risk of death [86].

Research by Freitas-de-Melo et al. [87] shows that primiparous ewes also exhibit a shorter period of receptivity and may reject foreign lambs intensely even two hours after birth using strong vocalisations and aggressive behaviour. In contrast, multiparous lambs are more tolerant of non-siblings, which may indicate a longer period of openness to bonding. Lambs from multiparous mothers burped less frequently before the first suckling, which may be a sign of greater vitality; however, they showed a higher heart rate in a separation test conducted at three months of age, suggesting a stronger stress response to separation from the mother.

Environmental factors may also play a significant role in bond formation, which can be significantly disrupted by the presence of other sheep, noise or stress in the first hours after birth [83], promoting rejection of the offspring. In sheep, care for the young is expressed not only in direct care but also as changes in foraging behaviour, with mothers adapting their actions to the needs of the lambs [88].

Sheep social behaviour, especially that of ewes, is also strongly dependent on their physiological state. During the periparturient period, ewes show more caution and maintain a distance from the flock, preferring to concentrate on their offspring. In contrast, during the grazing period, their behaviour becomes more synchronised with the rest of the group, indicating high social flexibility and adaptability [84].

### 3.4. Relationships with Humans

Sheep welfare is strongly dependent on their relationship with humans. Like other livestock, sheep are able to establish positive relationships with humans, which benefits their well-being [89,90,91]. One way to build such relationships is through habituation, whereby sheep become accustomed to the presence of humans; indeed, they have been found to demonstrate milder anxiety responses after repeated exposure to neutral or positive stimuli [91].

Sheep can also learn to associate the presence of humans with rewards, such as stroking or food, making them more willing to approach people offering such positive stimuli [91]. Animals tamed from a young age may show behavioural signs of attachment, such as seeking contact after a period of isolation [92]. Sheep can also recognise faces even after two years, indicating the permanence of their relationship [93].

Positive relationships are indicated by sheep voluntarily approaching humans, as well as by signs of relaxation, such as a lowered heart rate, dangling ears or ruminating in the presence of a human [94]. Human contact can also increase levels of oxytocin, the hormone associated with social bonds [95]. These relationships also appear to be influenced by various other factors, such as previous experience from youth, as animals socialised with humans from youth show less skittishness [92].

Sheep can form lasting and positive relationships with humans, which translates into greater wellbeing. It is therefore important for the keeper to pay attention to their comfort signals, such as approaching voluntarily, and allow them to choose when and how to interact with humans [89].

Sheep behaviour can generally be largely modified by human activity. The animals respond well to a calm but firm approach. They can sense the ability to recognise human intentions and appear to submit more readily to a handler they perceive as a leader. Generally,, sheep are believed to respond better to short, firm commands than to uncertain or chaotic behaviour [96].

### 3.5. Stereotypies in Sheep

Stereotypies are repetitive, unnatural oral behaviours that occur most often under intensive husbandry conditions, where animals have limited access to natural activities and exploration of the environment [97]. Under such conditions, behaviours such as bar-biting or slat-chewing are often observed [98]. Another more commonly recorded manifestation of stereotypy is wool-biting, which is particularly prevalent when the diet is poor in roughage or too rich in concentrates; this can limit chewing time and can lead to gastrointestinal discomfort [99]. As such, dietary composition plays an important role in exacerbating or alleviating these behaviours. Studies show that increasing the proportion of fibre can significantly reduce the incidence of undesirable oral behaviour. Vasseur et al. [99] found the addition of barley straw to sheep diets significantly reduced the incidence of wool biting.

## 4. Protocols for Evaluating Sheep Welfare

By using standardised sheep welfare assessment protocols, it is possible to perform systematic analysis of various aspects of welfare based on behavioural, physiological or environmental indicators. Five main protocols have been described in the literature [100,101,102,103,104]. While all are based primarily on animal-based indicators, some also incorporate resources and production data. Among these, the AWIN protocol stands out with its two-level structure, which reduces stress in animals and shortens the assessment time. In contrast, the Napolitano and Stubsjøen protocols place greater emphasis on integrating environmental parameters and resources, such as access to water and herd density. The Munoz protocols, on the other hand, have been adapted to extensive systems, allowing for the individual assessment of selected sheep and the monitoring of threats to their welfare (Table 3).

### 4.1. AWIN

In 2011, in response to the lack of a welfare assessment system for small ruminants in the Welfare Quality^®^ project, the Animal Welfare Indicators project was performed to develop the AWIN protocol [103,105,106]. The AWIN uses a two-level assessment comprising a quick overview of the entire herd (level one) and a detailed assessment of selected animals (level two). The first level is based on simple indicators that do not cause stress (Table 3), while the second is used when problems are detected or the farm is classed among the lowest 5% in the reference population. This approach is characterised by shorter assessment time and lower animal stress levels [105] (Table 3).

### 4.2. The Napolitano Protocol

This protocol, developed in Italy, uses a five-category approach based on the Animal Needs Index [107], with four focussing on resources and the fifth on animal-based indicators [101]. The characteristics assessed include body condition, skin changes, soiling, hoof overgrowth and lameness, as well as mutilations such as tail docking. Scores are assigned according to the frequency of occurrence of a given indicator in the herd and summed to give a final score [101] (Table 3).

### 4.3. The Stubsjøen Protocol

The Stubsjøen protocol was developed based on the Five Freedoms system for dairy cattle [102]. It includes 16 animal-based indicators, 15 resource indicators and three production indicators. The assessment procedure itself begins with observation of the herd, followed by a clinical examination of selected animals and an analysis of their behaviour in terms of anxiety and human–animal relations [102] (Table 3).

### 4.4. The Munoz Protocols

Munoz et al. [100,104] developed two protocols for extensive systems. The first comprises five welfare domains with 17 indicators, eight of which were selected for their reliability and feasibility. The second protocol focuses on six main indicators: body condition, fleece condition, skin lesions, tail length, dag score and lameness, and records the number of animals requiring care. These protocols allow the detection of welfare problems in extensive systems and indicate areas for improvement [100,104] (Table 3).

## 5. Indicators of Welfare in Sheep

Sheep welfare is assessed using a variety of physical, behavioural and environmental indicators. The most commonly used measures include lameness/gait score, body condition score, fleece cleanliness, faecal soiling/diarrhoea, tail length/mutilations, skin lesions/integument condition/skin irritation, fleece quality/fleece condition, familiar human approach/fear/flight distance, udder problems (mastitis or other udder problems) and hoof condition (hoof overgrowth/hoof condition). These indicators have been used in studies by Munoz et al. [100,104], Napolitano [101] and Stubsjøen [102], and as part of the AWIN system [105] (Table 3).

Various physiological and health parameters are used, such as the severity of panting, general body condition, skin changes, hoof condition, and eye and nose discharge [100,105], as well as behavioural indicators, such as isolation from the group, stereotypical behaviour, excessive scratching and reactions to human presence, such as flight distance (Table 3).

They also include environmental and management parameters, such as access to water and shelter, herd density and lamb mortality, which reflect the impact of husbandry practices on animal welfare [101,102]. In addition, general indicators, including mucous membrane colour, rumen fill, presence of oedema or ear damage, allow for a comprehensive assessment of health and comfort [100,105] (Table 3).

The welfare assessment protocols that focus on animal-based indicators, i.e., outcome measures that reflect the actual experiences of animals, offer a direct indication of how animals feel in a given environment and how they function. Hence, they are considered more reliable than assessments based on resources or the environment [108].

### 5.1. Body Condition Score (BCS)

A widely used nutrition-based indicator of sheep welfare is their body condition, determined by the body condition score (BCS) [105,109]. Briefly, the score reflects the amount of fat and muscle covering the backbone, which in turn is indicative of the balance between energy intake and expenditure in the sheep. The BCS is considered an indicator of nutritional status, feed intake motivation and overall physiological condition of sheep, and its values correlate with reproductive performance, lactation and disease resistance [105,109].

Body condition is assessed individually, usually by palpation of the lumbar spine. The Russell et al. [109] scale distinguishes four categories: cachexia (BCS ≤ 1.0), lean (<2.0), fit (2.0–4.0) and fat (>4.0), with each category reflecting the degree of nutrition and nutritional welfare status of the animal [105,109].

The BCS has demonstrated convergence with other indicators of biological function, such as health, fertility and mortality [110], and correlates with certain indicators of tissue mobilisation, i.e., the levels of non-esterified fatty acids and glucose [111]. Such assessment has practical significance for welfare as lean animals have been shown to have a stronger motivation for food intake and a higher risk of gestational toxaemia [111,112].

The animal should undergo regular monitoring for body condition, as this is an important welfare indicator than can be used to detect malnutrition, overfeeding or unequal access to feed. A low BCS indicates inadequate nutrition, disease or parasites, while a high BCS indicates overfeeding, limited activity and risk of metabolic disorders. Thus, BCS assessment can support informed herd management by integrating various nutritional, health and behavioural aspects [105] (Table 4).

### 5.2. Wool Cleanliness

Another important indicator of sheep welfare is the condition of the fleece, which is related to their degree of comfort when resting. The presence of dry, clean wool suggests that the animal has had access to a dry and comfortable place to lie down, while excessive soiling may indicate stress, health problems or inappropriate environmental conditions [105].

Wool cleanliness is assessed on a five-point scale: 0 indicates a clean and dry sheep, with no signs of dirt or contamination; 1—a dry or slightly damp animal, with minor soiling resulting from weather conditions or daily handling in the pen; 2—very damp or wet, with mud or pasture faeces; 3—very wet, heavily soiled with mud or faeces; 4—extremely dirty, very wet, covered with mud or faeces, including on the face and back [100,105] (Table 4).

### 5.3. Lameness

Lameness is an abnormality in sheep movement caused by pain and a limited ability to bear weight on the affected limb. It most often caused by hoof infections and can affect both lambs and adult sheep. As such, it is an important indicator of welfare [113,114]. Assessment can be carried out individually or in groups, with group observation being easier and allowing more cases to be detected [115]. The most practical and reliable method is a binary classification of “healthy” vs. “lame”; the AWIN protocol uses a four-point scale: 0—no lameness; 1—mild, with shortened stride and head swaying; 2—moderate, with pronounced head swaying and limited weight bearing on the limb; 3—severe, with the animal not standing or bearing weight on the affected limb [105]. Lameness is widely recognised as a reliable indicator of sheep welfare and is used in all major animal assessment protocols (Table 4).

### 5.4. Access to Water and Shelter

Access to water is a very important indicator of sheep welfare, and one that corresponds to the criterion of “no prolonged thirst” [105,108]. The assessment includes confirming the presence of drinking points, and checking their functioning, availability and cleanliness; these can be buckets, troughs, automatic drinking fountains or natural water sources. The indicator can be recorded as “yes/no” or as a percentage of animals with access to water; this allows a ready assessment of whether the basic physiological needs of the flock are being met.

In addition, access to shelter is an important indicator of sheep welfare, corresponding to the criterion of “protection from adverse weather conditions” [105]. The assessment includes confirming the presence, availability and actual use of natural or artificial shelters. The indicator can be recorded as ‘yes/no’ or as a percentage of animals with access, allowing an assessment of whether sheep are protected and their physical and behavioural welfare is maintained (Table 4).

### 5.5. Lamb Mortality

Lamb mortality serves as an effective indicator of sheep welfare as it reflects both the physical condition of the ewes and the quality of flock management [116,117,118]. An increased number of neonatal deaths may indicate maternal malnutrition, disease, stress or inappropriate breeding practices [119,120]. While mortality alone may not indicate the exact cause of a problem, high mortality can serve as an early warning signal for other potential animal welfare deficits [132] (Table 4).

### 5.6. Lying Time

Lying time reflects the extent to which sheep are able to rest in comfortable and safe conditions [121,122]. It can be reduced by lack of space, inadequate flooring or social stress, and lengthened by improved living conditions [123,124]; it is also influenced by physiological factors, such as pregnancy and health (e.g., lameness and scabies) [125,133,134]. While rest time is strongly related to the circadian rhythm among sheep kept in pasture conditions, those in closed systems spend most of the day lying down [134] (Table 4).

### 5.7. Breech Soiling (Dag Score)

Sheep may demonstrate soiling of the wool around the tail and hind legs, caused by faecal matter sticking to the coat; this is often associated with the presence of gastrointestinal parasites and diarrhoea [126]. Its severity is influenced by inter alia wet pastures, incorrect deworming and poor animal condition [127]. The degree of soiling can be assessed quickly and visually, without physical contact with the animal, with high consistency between assessors [128]. The severity of soiling is determined on a five-point scale: 0—no soiling, 1—slight traces of faeces, 2—clumps around the anus, 3—soiling reaching the tail and upper legs, 4—extensive soiling up to the hocks [105]. Higher values (3–4) indicate poor hygiene and an increased risk of fly strike; as such, this indicator is an important element in assessing sheep welfare. Although the result may be partly dependent on the type of feed and seasonal conditions, this parameter is considered useful in monitoring welfare (Table 4).

### 5.8. Lying Synchrony

Lying synchronisation describes the extent to which sheep rest simultaneously. For a flock to rest together, there must be sufficient space for there to be no competition [135]. When the flock density is too high, simultaneous resting is restricted and animals become more restless [121,122]. Close synchronisation of resting behaviour indicates good welfare and stable relationships within the flock (Table 4).

### 5.9. Mastitis and Udder Lesions

Mastitis and changes in the udder are also significant welfare indicators, as the presence of inflammation or lumps indicates pain and discomfort and can affect lamb feeding and overall health. The assessment is carried out by palpation in lactating ewes, according to a tripartite score system: 0—no changes, udder soft and elastic, 1—mild changes, single small lumps or slight hardness (<10 cm), 2—severe changes, lumps on both sides of the udder or a large lump (>10 cm). This indicator is easy to assess when handling animals and allows for the rapid detection of both acute and chronic health problems, making it a reliable tool for monitoring welfare [105] (Table 4).

### 5.10. Respiration Quality

The respiratory quality index assesses how easily a sheep breathes and the presence of coughing, audible breathing sounds or nasal discharge. Assessment requires the animals to be handled, during which their head is stabilised and their breathing observed. It is scored in two categories: 0—no problems, breathing freely, no coughing or discharge, and 1—respiratory problems present, which allows for an assessment of breathing comfort and respiratory health [105] (Table 4).

### 5.11. Social Withdrawal

Social withdrawal occurs when sheep isolate themselves from the flock and do not respond to stimuli from their surroundings; this may indicate stress, illness or general discomfort as healthy sheep typically rest, chew their cud and graze in synchrony with other members of the flock, while remaining alert to changes in their environment. The assessment involves observing the flock for a specified period of time, during which the number of individuals that remain alone and do not participate in group behaviour are counted. The observation is scored based on the number of withdrawn individuals: 0—no socially-withdrawn animals, 1—a small number, 2—a moderate number, 3—a large number; the final score indicates the overall level of social withdrawal in the flock [105] (Table 4).

### 5.12. Stereotypies

Stereotypies are repetitive movements, such as walking along the same route, climbing or head banging, and repetitive oral activities (e.g., licking, biting, pulling the wool of other sheep or parts of the pen). These do not typically occur in healthy animals, and are mainly observed in sheep kept indoors, in confined spaces or on a diet low in fibre. They are assessed by observing the flock for a specified period of time and counting the number of individuals exhibiting such behaviours; this score is then used to determine the overall level of abnormal behaviour in the group [105,129,130,131] (Table 4).

### 5.13. Fleece Condition

The condition of the fleece can also play an important part in assessing welfare, as it allows for the detection of hair loss and baldness, as well as the presence of external parasites, which can affect the health and comfort of animals. In the AWIN protocol, fleece condition is assessed on a three-point scale: a score of 0 indicates a fleece in good condition, with no lumps or signs of external parasites found after separation; a score of 1 indicates slight coat loss, minor flaking or bald patches up to 10 cm in diameter, with the coat possibly showing lumps, dandruff or minor signs of parasites; a score of 2 indicates significant coat loss, with bald patches larger than 10 cm in diameter and clear signs of external parasites [100,105].

Regular assessment of fleece condition allows effective monitoring of skin health and fleece quality, as well as the early detection of health problems and unfavourable environmental conditions (Table 4).

### 5.14. Qualitative Behaviour Assessment (QBA)

QBA allows an assessment of emotional state by observing the behaviour and posture of the sheep, and its interaction with the environment. The assessment is based on certain characteristics known to reflect the emotional well-being of the animals, such as activity, calmness, curiosity and tension. Observations are made at selected points in the flock, after the animals have become accustomed to the presence of the observer, and each observation is scored on a continuous visual scale: extreme values indicate the absence or full expression of a given trait in the flock, while intermediate values indicate the degree of its presence [105] (Table 4).

### 5.15. Familiar Human Approach Test—Human Relations Assessment

The test assesses how sheep react to the approach of a familiar person (e.g., a farmer) by measuring the distance at which the animals demonstrate escape behaviour or a willingness to voluntarily interact by sniffing or approaching. A shorter flight distance or active approach by the sheep indicates a good relationship with humans and positive welfare [105] (Table 4).

## 6. Transport and Slaughter of Sheep

### 6.1. Sheep Welfare During Transport

Transport entails a number of factors which represent a significant source of stress for sheep [136]. These include environmental factors, such as changes of surroundings, limited movement, noise, changes in temperature and humidity, and inadequate ventilation [137,138]. They are also subject to physiological factors such as limited access to water and feed, fatigue, and stress resulting from contact with unfamiliar individuals [137,139]. In addition, there are various health-related factors, such as the risk of injury, bruising, mutilation and metabolic disorders, as well as reduced immunity [140,141] (Table 5).

Sheep experience fatigue during transport due to the interaction of various physical, psychological and environmental factors [170,171]. Fatigue itself is exacerbated by both physical exertion and emotional stress caused by, inter alia, noise, vibrations, crowding or contact with unfamiliar individuals. Prolonged stress, fear and anxiety lead to mental exhaustion, as also noted in human studies [172,173] (Table 5).

Interviews with animal welfare experts and representatives of the transport industry indicate that the perception of fatigue in sheep is often shaped by the natural tendency of humans to anthropomorphise their experiences. The respondents compared the behaviour of sheep to their own feelings, noting, inter alia, that the stress of being in new groups or unfamiliar environments can lead to mental fatigue as a result of sensory overload [174,175]. However, experts warn against assuming that sheep react in an identical way to humans during such situations; such anthropomorphisation may result in inadequate transport practices, e.g., in terms of vehicle ventilation or watering systems [171,176].

It is often difficult to assess the condition of sheep, as they tend to mask signs of fatigue until they reach a critical point. It is therefore recommended to use more holistic assessments encompassing all aspects of their life and behaviour, including qualitative methods of behaviour observation, such as Qualitative Behaviour Assessment (QBA). Such focussed approaches can highlight subtle changes in the interaction of sheep with their environment and provide a more accurate picture of their fatigue and overall welfare [177,178,179,180].

Symptoms of fatigue may include apathy, reduced mobility, balance problems, increased need to lie down, or reduced chewing. However, their interpretation should take into account the environmental context and individual differences between animals [170,181].

The need to maintain their balance during vehicle movement and constant tension in the limbs, especially in confined spaces with high densities of other animals, is a source of physical fatigue [136,140,181]. This is exacerbated by lack of water and feed during long-term transport, leading to further energy depletion, dehydration and weakened immunity [182]. The level of fatigue is also significantly influenced by the sensory overload resulting from excessive noise, vibrations and odours, to which sheep are particularly sensitive [174,175,183,184] (Table 5).

Fatigue is primarily influenced by journey quality, which is influenced by journey time, driving style, microclimate, vehicle type, number of stops, and access to water and feed. Long journeys in unsuitable conditions result in the accumulation of stressors and a deterioration in welfare [170,182] (Table 5).

However, fatigue is also determined to some extent by individual characteristics of the animals. Age, condition, temperament and personality type influence their ability to cope with stress and the demands of transport [137,147,182]. In addition, previous transport experiences and breeding conditions play a role: individuals accustomed to gentle transport cope better, while those without experience or with negative experiences show stronger stress reactions [147,185].

According to current EU regulations [186], sheep transport may last a maximum of 29 h for adult animals and 19 h for unweaned lambs, after which a 24-h rest period with unloading is required [170]. However, research indicates that, inadequate transport conditions can nevertheless lead to the accumulation of stressors and marked fatigue, even when these limits are respected [182,187].

### 6.2. The Impact of Slaughter Conditions on Sheep Welfare

The stages preceding slaughter, such as the transport, unloading and movement of the sheep to the slaughterhouse, have a significant impact on their welfare. Inappropriate transport conditions, excessive or insufficient temperature, fatigue, lack of access to water and feed, and improper handling are significant stressors, indicated by increases in the levels of stress hormones, glucose and lactate in the blood. These physiological responses indicate HPA and SAM axis activity and may negatively affect meat quality by reducing muscle glycogen stores [167,188].

At the slaughterhouse, inappropriate handling of sheep, such as pulling them by their wool or lifting them by their limbs, increases stress, pain and the risk of injury, which also significantly reduces animal welfare. The animals respond to such treatment with characteristic stress behaviours such as immobility, changes in head position, body swaying and high-frequency bleating [164,189].

Most threats to sheep welfare during slaughter result from human error, which typically stem from inexperience or fatigue among staff. It is therefore important to provide suitable training for employees, ensure that the process is supervised, follow standard procedures and use appropriate equipment to reduce stress and suffering for the animals [164].

Stunning is performed using mechanical (e.g., captive bolt pistols) or electrical methods. Effectiveness depends on the correct setting of the device and the experience of the employee: errors at this stage can cause excessive suffering as a result of incomplete stunning. Bleeding should be carried out immediately after stunning to prevent the animal from regaining consciousness [165,166,167,168,169].

Both transport conditions and pre-slaughter handling also influence animal welfare and meat quality. Pre-slaughter stress leads to the depletion of energy reserves, particularly muscle glycogen; as a result, glucose and lactate are released into the blood, resulting in the production of lower-quality dark meat with higher pH (>5.74) [188]. In addition, noise, poor road conditions and driving style further reduce muscle glycogen content, increasing stress and cortisol levels, and impairing meat colour and tenderness [142]. Similarly, a lack of ventilation in the vehicle can lead to heat stress and further increase muscle pH [146], while long transport promotes dehydration, weight loss and reduced carcass yield [154].

During transit, sheep are subject to further behavioural stress due to contact with new environments or unfamiliar animals [155], and metabolic stress, accompanied by a decrease in glycogen level, due to lack of water and feed [156]. Pre-slaughter procedures, such as catching and stunning, can also impair meat quality by causing injury and pain [159]. Even changes in altitude above sea level can increase oxidative stress and affect hormonal parameters [142]. All these factors together have a negative effect on sheep welfare and reduce the quality of the meat obtained (Table 5).

## 7. Production Systems, Welfare and Technology

Different sheep farming systems have varying degrees of access to technology, which also directly affects animal welfare. For example, farms equipped with modern devices such as automatic feeders, drinking points, environmental sensors, and activity and health monitoring systems, can quickly detect problems with the animals and maintain optimal living conditions. However, on those with limited access to such solutions, the animals are more likely to experience poorer conditions and be more vulnerable to stress and disease.

Sheep kept in an extensive system mainly graze on pastures and open areas, where access to modern tools is minimal. The farmer observes the animals mainly visually and performs routine checks. However, this lack of automation and sensors makes the animals more vulnerable to stress and health problems resulting from adverse weather conditions and limited access to feed and water [7] (Table 6).

Sheep can also be kept in an indoor system, consisting of enclosed or partially enclosed spaces, which can be equipped with devices intended for improving the comfort and overall welfare of the flock. This equipment allows the farmer to better monitor the health and behaviour of the animals and respond more quickly to stressors or health problems [7,190,191,192,193,194,195] (Table 6).

Intensive systems are typically characterised by a high level of automation and make extensive use of technology: automatic ventilation systems, temperature and humidity sensors, automatic feeders and drinkers, health monitoring and data analysis using artificial intelligence. Again, this constant monitoring allows for early detection of disease and stress, ensuring the highest possible level of welfare for large numbers of individuals [7,196,197,198] (Table 6).

### Sheep Welfare and Modern Technology

In extensive systems, the sheep are increasingly benefitting from the use of modern digital technology, which allows continual monitoring of individual animals. Electronic identification (EID) allows each animal to be tracked individually, with this information being linked to GPS systems, motion sensors and automatic weighing systems. This approach, known as Precision Livestock Farming (PLF), allows for better herd management, increased work efficiency and profitability and, above all, improved animal health and welfare through early detection of health problems and stress [199,200,201,202] (Table 7).

It has been found that a PLF system can enable effective monitoring of sheep behaviour, lambing, lameness and body weight; it also allows real-time assessment of animal welfare, which supports a quick response to changes in the environment and animal health [211,212].

In indoor farming, increasing use is being made of modern digital technology for Thanks to electronic identification, each animal can be tracked individually, with further data being obtained through various other devices such as accelerometers, automatic drinking and feeding systems, visual monitors and biological sensors [209] (Table 7). These solutions allow precise flock management, including monitoring of behaviour and body weight, calving or lameness symptoms, and to assess animal welfare. This allows the farmer to respond quickly to adverse environmental conditions or health problems, improving the animals’ quality of life and reducing the risk of disease [209].

In the indoor system, the building is equipped with ventilation systems and temperature and humidity sensors, which, in combination with identification electronics and motion sensors, enable continuous monitoring of environmental conditions and animal welfare [198].

The intensive system also employs a range of automated technologies, such as automatic feeding and watering systems, microclimate control, ventilation systems, and animal health and activity monitoring systems (Table 7). The data collected from these devices can be analysed using digital tools and artificial intelligence (AI); this can be used to monitor animal welfare and provide an early indication of potential health problems [197,198].

The use of these technologies makes it possible to maintain optimal environmental conditions and reduce animal stress, even with a large number of animals in the system. It can also improve animal health, welfare and production efficiency by facilitating rapid responses to emerging health threats.

## 8. Conclusions

Sheep welfare is a complex issue involving physical, psychological, social and environmental aspects. The well-being of the animals is influenced by housing conditions, feeding, access to water, grooming and social interactions within the flock. It is very important to be able to perform natural behaviours and to reduce stressors. The choice of rearing system (intensive, extensive) also has a direct impact on the level of welfare of the sheep. In this regard, protocols incorporating both environmental and animal-based indicators are of particular value, as they allow for a comprehensive assessment of sheep welfare.

A review of the available literature confirms that both extensive and intensive systems can have positive and negative impacts on the welfare of sheep. The extensive system allows the sheep to behave naturally, but exposes them to variable environmental conditions, such as the risk of drought or exposure to parasites. In contrast, the intensive system provides better health and nutritional control, but limits space and promotes behavioural disorders.

It was also found that confined systems should be enhanced with environmental enrichment, such as bedding and appropriate feed, to meet the need for exploration and foraging. Such enhancements are essential for satisfying natural needs of the sheep: access to pasture reduces stereotypic behaviour and improves psychological well-being.

Regarding feeding, roughage is important in the diet, as a shortage is associated with digestive disorders and frustration. In addition, due to the hierarchy within the flock, feeding stations should be designed in such a way as to limit competition between individuals. Lambs should be weaned from their mothers gradually to minimise the associated stress. Grooming procedures, such as shearing and hoof correction, must be carried out in a timely manner and with minimal stress to the animals. Trimming the hooves should be performed with the animal standing, as this is less stressful than lying down. Social bonds, both between sheep and with humans, are fundamental to good welfare. Sheep are able to recognise carers and respond better to gentle, regular contact.

Stereotypic behaviours such as rod chewing or wool chewing are indicative of poor welfare and result from inadequate environmental stimulation or poor nutrition. They can be reduced by increasing the proportion of fibre in the diet.

In general, by implementing comprehensive husbandry standards, and respecting behavioural, environmental and health aspects, it is possible to significantly improve sheep welfare.

## Figures and Tables

**Table 1:** Differences between extensive and intensive systems for sheep farming.

Criterion	Extensive System	Intensive System
Environment and space	Open grazing land, often large acreage	Enclosed or semi-enclosed rooms, limited space
Freedom of movement	High—free movement	Restricted—within the pen only
Nature of the environment	Natural, variable, stimulus-differentiated	Homogeneous, predictable, stimulus-poor
Thermoreglation	Dependent on weather and natural shelters	Controlled microclimate (buildings, ventilation)
Feed availability and nutrition	Forage (grass, hay); risk of malnutrition during drought and other natural disasters	Concentrated feed, controlled feeding; less risk of deficits
Stocking density	Low—dispersed animals	High—groups in a closed environment
Human contact	Occasional (mainly zootechnical/veterinary treatments)	Daily and intensive (feeding, milking, checks, etc.).
Health risks	Higher risk of parasites (grazing)	Greater risk of infectious diseases at high densities
Ability to express natural behaviour	Greater—exploring, foraging, avoiding contact	Limited—less stimulus and space
Response to extreme conditions	Adaptable (approaching, using shade), but risk of hypothermia or heat stress	Reduced risk due to microclimate control, but less opportunity for behavioural adaptation
Social relations and links	Development of natural structures (mother-calf bonds, subgroups, sexual segregation)	Disrupted by separation, isolation, group homogeneity
Hierarchy and domination	Subtle, stable, fewer conflicts; domination mainly by resources	More pronounced, increased agonist interactions at high density
Coordination of herd movements	Natural, leaders initiate movement, regardless of dominance	Often artificially directed; spatial constraints reduce spontaneity
Communication and recognition	Based on visual, olfactory and auditory cues; recognition of individuals and groups	Potential for distortion by density and environmental homogeneity
Social stress and isolation	More stable relationships, opportunity for eye and physical contact	Higher risk of isolation stress, elevated cortisol levels
Aggressive behaviour	Rare—subtle communication and group stability	More frequent—conflicts at the feeding area, social pressure
Responses to environmental change	Possible stress on relocation (e.g., seasonal grazing)	Better stability of conditions but less adaptability
Reproductive behaviour	Natural selection of partners, free sexual behaviour	Controlled reproduction (insemination, synchronisation), possible behavioural frustration
Social well-being	Higher—natural interactions possible, but dependent on resource availability	Limited—isolation, mixing of groups, social frustration
Spatial relationships	Individuals maintain personal and social distances; possible subgroups	Smaller distances, limited adjustability
Risk of behavioural disorders	Smaller—more exploratory behaviour, less stereotyping	Greater—stress, frustration, disruption of social relationships

**Table 2:** Sheep welfare in extensive and intensive systems in the context of the Five Freedoms.

Five Freedoms	Description in the Context of Sheep	Impact of the Extensive System	Impact of the Intensive System	Welfare-Enhancing Practices	Authors Cited
1. Freedom from hunger and thirst	Ensuring adequate quality and quantity of feed and constant access to water	Pastures provide natural access to grass and hay; risk of shortages during dry periods	Controlled feeding of concentrate mixes; less risk of deficits but no choice of feed	Regular monitoring of feed quality, provision of fresh water, supplementation of elemental deficiencies during critical periods	[1,22]
2. Freedom from discomfort	Appropriate environmental conditions, shelter, thermal comfort	Natural environment, adaptability, but risk of extreme conditions	Microclimate control, ventilation but limited space	Access to shelter and shade, introduction of litter, temperature control in buildings	[23,24]
3. Freedom from pain, injury and disease	Disease and injury prevention; preventive treatments	Risk of parasites and environmental diseases	Risk of infectious diseases with high stocking rates, better prevention	Regular grooming (shearing, hoof correction), vaccinations, health checks, minimising stress during treatments	[25,26]
4. Freedom to express natural behaviour	Ability to move, forage, interact socially	High degree of freedom of movement, natural hierarchies and social ties	Limited space and stimuli, controlled reproduction, less interaction	Environmental enrichment, provision of grazing land, maintenance of social groups, opportunities for exploration and play	[27,28]
5. Freedom from fear and stress	Providing a sense of security, reducing stress	Low risk of human stress, greater control over own behaviour	Frequent human contact and high density—higher risk of stress	Gradual introduction of treatments, gentle methods of weaning lambs, avoiding excessive isolation, respecting hierarchy	[29,30]

**Table 3:** Comparison of selected protocols for assessing sheep welfare.

Protocol	Range of Indicators	Indicator Type	Assessment Method	Practical Points	References
AWIN (2011)	Lameness, BCS, fleece condition, tail length, skin lesions, panting, social withdrawal, QBA, mastitis, hoof condition, faecal soiling	Animal-based	Two levels: rapid flock assessment + detailed individual assessment	Minimal stress for animals. Assessment can be quick (level 1) or more detailed, in the case of health problems or low herd score (level 2)	[105]
Napolitano (2009)	BCS, integument alterations, animal dirtiness, hoof overgrowth, lameness, lesions, mutilations	Animal and resource-based	Scoring by frequency of problems in the flock	Takes into account both animals and resources; recommends a visit after shearing to better assess skin and coat condition	[101]
Stubsjøen (2011)	Lameness, BCS, skin lesions, fleece condition, human–animal interaction, respiratory signs, production data	Animal-, resource- and production-based	Herd observation + clinical examination of 10 random sheep + analysis of production data	Inter-observer reliability high, requires visits indoors, assessment of behaviour and resources	[102]
Munoz (2018)	BCS, fleece condition, skin lesions, tail length, dag score, lameness, flight distance	Animal-based	Individual evaluation of selected animals in the flock	Designed for wide area systems, tested in different phases of the pro-duction cycle	[100]
Munoz (2019)	BCS, fleece condition, skin lesions, tail length, dag score, lameness, number of animals requiring care	Animal-based	Individual sheep assessment + registration of animals in need of care	Adaptation of 2018 protocol, includes fewer indicators, suggests inclusion of governance and environmental conditions	[104]

**Table 4:** Welfare assessment indicators in sheep.

Indicator	Parameter	Assessment Method	Scale/Scoring	Significance for Welfare	References
Body Condition Score (BCS)	Amount of body fat and muscle, nutritional status	Palpation of the lumbar spine	0–1: depleted, <2: lean, 2–4: in good shape, >4: fatty	Informs on nutrition, reproduction, immunity and phycological condition	[105,109,110,111]
Wool cleanliness	Hygiene and comfort while resting	Visual observation	0–4 (clean to extremely dirty)	Indicates stress, illness, environmental conditions	[100,105]
Lameness	Pain and restriction of movement	Individual or group observation	0–3 (none to severe)	Indicates health problems and influence on animal comfort	[105,113,114,115]
Access to water	Fulfilling physiological needs	Monitoring water points	Yes/No or % of animals with access	Prevents chronic thirst	[105,108]
Access to shelter	Protection against adverse weather conditions	Monitoring the presence and use of guards	Yes/No or % of animals with access	Ensures physical and behavioural comfort	[105]
Lamb mortality	Quality of flock care and condition of dams	Analysis of breeding records	Number of deaths or %	High mortality signals malnutrition, disease or stress	[116,117,118,119,120]
Lying time	Opportunity to relax	Observation of resting behaviour	Number of minutes per day/% of animals lying down	Reduced time indicates stress, lack of comfort or inadequate conditions	[121,122,123,124,125]
Breech soiling (Dag score)	Hygiene of the tail area, presence of parasites	Visual observation	0–4	Indicates health problems, hygiene and the risk of mu-cha infestation	[105,126,127,128]
Lying synchrony	Simultaneous resting of the flock	Group observation	% of sheep lying at one time	High synchronisation score indicates good welfare and adequate space	[101,121,122]
Mastitis and change sin the udders	Pain, discomfort and health conditions	Palpation of lactating sheep	0–2	Rapid assessment of health problems and welfare of lactating sheep	[105]
Respiration quality	Ease of breathing and respiratory health	Observation of breathing when working with the animal	0–1	Indicates respiratory diseases and stress	[105]
Social withdrawal	Level of integration with flock, response to stimuli	Observation of the flock	0–3 (none to a large number withdrawn)	High levels of withdrawal indicate stress or illness	[105]
Stereotypie	Abnormal repetitive behaviour	Observation of the flock	Number of animals showing stereotypes	Indicates stress, limited space or poor diet	[105,129,130,131]
State of the fleece	Hair loss, alopecia, external parasites	Visual observation	0–2	Assessment of skin health and fleece quality	[100,105]
Qualitative Behaviour Assessment (QBA)	Emotional state of the sheep	Observation of behaviour, attitude, interactions	Continuous VAS scale	Assesses the emotional well-being and behavioural style of animals	[105]
Familiar Human Approach Test	Human relations	Observing the sheep’s reaction to a familiar human approaching	Escape distance in metres; active approach	A short distance or active approach by the sheep indicates positive relationships and well-being	[105]

**Table 5:** The impact of transport and pre-slaughter stressors on sheep welfare and meat quality.

Process Stage/Stressor	Effect on Sheep Welfare	Effect on Meat Quality	References
Transport (poor road quality, noise, driving style)	Stress, anxiety, fatigue, elevated cortisol levels	Reduction in muscle glycogen → lower lactic acid production → impaired meat colour, texture and flavour	[140,142,143,144,145]
Transport (vehicle design, ventilation)	Heat or cold stress, risk of suffocation in the absence of ventilation	Increased muscle pH, reduced meat quality	[146,147,148,149,150]
Transport (journey length)	Fatigue, hunger, thirst, weight loss	Decreased carcass yield, reduced water retention capacity, higher muscle pH	[150,151,152,153,154]
Transport (new environment, new animals)	Behavioural stress, adjustment difficulties	Possible effect on muscle metabolism and meat quality.	[146,155]
Transport (lack of water and feed)	Dehydration, hunger, metabolic stress	Muscle glycogen depletion → reduced lactic acid production	[154,156]
Unloading and handling, pre-slaughter procedures	Anxiety, pain, fatigue, physical injuries	Decrease in muscle glycogen → lower lactic acid production → deterioration in colour, flavour and tenderness of meat	[157,158,159,160,161,162,163]
Slaughterhouse work (pulling wool, lifting)	Stress, pain, injuries, behavioural reactions: immobility, body swaying, head raising/lowering, high-frequency bleating	Possible muscle damage and poorer meat quality	[164]
Stunning	Incomplete stunning, stress, further suffering	Delayed muscle fixation, elevated pH	[165,166,167,168,169]
Bleeding	Delay after stunning → regaining consciousness, pain	Dark meat, higher pH	[165,166,168]
Height above sea level	Oxidative stress, hormonal changes (T3, tyrosine, MDA)	Possible effect on muscle metabolism and meat quality.	[142]

**Table 6:** The impact of applied technologies on welfare in different sheep housing systems.

Housing System	Technology/Tools	Effect on Welfare
Extensive	Minimal access to technology; visual observation and routine checks	Welfare compromised by weather-related stress, limited access to feed and water, and health issues
Indoor	Automatic feeders and drinkers, activity sensors, video monitoring, environmental sensors	Enables better control of animal health and behaviour; faster response to stress and health problems, improved quality of life
Intensive	Automatic ventilation systems, temperature and humidity sensors, automatic feeders and drinkers, health monitoring, data analysis using AI	Continuous monitoring of conditions and health, early detection of disease and stress; ensures optimal living conditions and the highest level of welfare even in large flocks

**Table 7:** Modern technologies used in sheep farming and their impact on animal welfare.

Housing System	Technology	Effect on Animal Welfare	References
Extensive	GPS collars, activity sensors, video monitoring, drones, automatic waterers	Promotes natural behaviour, reduces stress, supports physical and mental health	[199,200,203,204,205,206]
Indoor	Accelerometers, automatic drinkers and feeders, ventilation systems, temperature and humidity sensors, video monitoring and biological sensors	Reducing stress resulting from confinement in buildings, early detection of diseases, activity assessment, health support	[198,207,208,209,210]
Intensive	Automatic feeding and watering, microclimate control, ventilation systems, health and activity monitoring, data analysis and AI	The ability to quickly detect diseases, monitor environmental conditions, and reduce stress in large numbers of animals	[197,198]

## Data Availability

Not applicable.
